# Preclinical assessment of combination therapy of EGFR tyrosine kinase inhibitors in a highly heterogeneous tumor model

**DOI:** 10.1038/s41388-022-02263-4

**Published:** 2022-03-19

**Authors:** Hiroshi Ikeuchi, Takeshi Hirose, Masachika Ikegami, Kazuya Takamochi, Kenji Suzuki, Hiroyuki Mano, Shinji Kohsaka

**Affiliations:** 1 Division of Cellular Signaling, National Cancer Center Research Institute, Tokyo, 104-0045 Japan; 2 Department of General Thoracic Surgery, Juntendo University School of Medicine, Tokyo, 113-8431 Japan; 3 Department of Musculoskeletal Oncology, Tokyo Metropolitan Cancer and Infectious Diseases Center Komagome Hospital, Tokyo, 113-8677 Japan

**Keywords:** Non-small-cell lung cancer, High-throughput screening

## Abstract

The development of tyrosine kinase inhibitors (TKIs) has improved the treatment of non-small cell lung cancer (NSCLC) with epidermal growth factor receptor (EGFR) mutations. The current research priority is to provide viable treatments for patients who have drug-resistant *EGFR* mutations. We evaluated the drug sensitivity of various EGFR mutants to monotherapies and combination therapies of EGFR-TKIs. In vitro, the transforming potential and drug sensitivity of 357 EGFR variants were assessed. In vivo, we tested the sensitivity of EGFR variants to different regimens of EGFR-TKIs by examining changes in the proportion of each variant within the tumor. Out of 357 variants thoroughly examined for transforming activities, 144 (40.3%) and 282 (79.0%) transformed 3T3 and Ba/F3 cells, respectively. Among the latter variants, 50 (17.7%) were found to be resistant or only partly resistant to osimertinib or afatinib. Four of 25 afatinib-resistant variants (16%) were sensitive to osimertinib, whereas 25 of 46 osimertinib-resistant variants (54.3%) were sensitive to afatinib. Despite the lack of a synergistic impact, TKI combination treatment effectively reduced in vivo the heterogeneous tumors composed of 3T3 cells with different EGFR variants. Regimens starting with afatinib and subsequently switched to osimertinib suppressed tumor development more efficiently than the opposite combination. Combination EGFR-TKI treatment may decrease tumor growth and prevent the development of resistant variants. This work created an experimental model of a heterogeneous tumor to find the best combination therapy regimen and proposes a basic notion of EGFR-TKI combination therapy to enhance the prognosis of NSCLC patients.

## Introduction

Lung cancer has the greatest death and morbidity rates among malignant tumors worldwide [1]. Non-small cell lung cancer (NSCLC) accounts for 75–80% of all lung cancers, and *EGFR* activating mutations are found in ~10–20% of advanced NSCLC cases in Europe and 30–50% of cases in East Asia [2–4].

Epidermal growth factor receptor (EGFR) tyrosine kinase inhibitors (TKIs) are the established first-line care against advanced NSCLC carrying sensitizing *EGFR* mutations, and have a considerable higher efficacy than the standard chemotherapy. Three generations of EGFR-TKI are now clinically available; the first-generation gefitinib and erlotinib, the second-generation afatinib and dacomitinib, and the third-generation osimertinib. Recent randomized controlled studies have consistently shown that second- and third-generation TKIs are more effective than first-generation TKIs [5–7]. Most recently, osimertinib demonstrated an overall survival advantage over first-generation TKIs, as well as a more favorable tolerability profile, establishing it as a standard first-line treatment option for common sensitizing *EGFR* mutations which are exon 19 deletions and the L858R mutation [8].

However, in many situations, determining the best TKI for a given EGFR mutant remains a difficult task. A critical research goal is to clarify the best effective way to treat patients with so-called rare *EGFR* mutations [9]. Uncommon somatic mutations are observed in ~20% of *EGFR* mutation-positive NSCLC, and represent a very diverse collection of genetic abnormalities within exons 18–21 [10].

So far, all head-to-head studies comparing various EGFR-TKIs have been limited to the most prevalent *EGFR* mutations. Even though LUX-Lung 3 and LUX-Lung 6 compared afatinib and chemotherapies in all *EGFR* mutation types [11–13], and a phase II study of osimertinib for rare mutations [14], there is insufficient prospective clinical evidence regarding the effectiveness of EGFR-TKIs against uncommon mutations. As a result, it is extremely desirable to develop a suitable strategy for treating NSCLC with rare mutations.

Another concern that must be addressed is the development of osimertinib resistance. Despite osimertinib’s powerful clinical efficacy, patients eventually acquire secondary resistance to it, posing a substantial problem given the scarcity of post-osimertinib therapy alternatives available to date [15, 16].

Considering that osimertinib resistance generally includes several mechanisms such as activation of alternative growth pathways or aberrant downstream signaling, osimertinib-based combination treatments are being studied intensively.

Combination medicines for osimertinib include inhibitors of downstream signaling pathways [17], inhibitors of multiple signaling pathways [18–22], angiogenesis inhibitors [23], immune checkpoint inhibitors [24], and cell cycle-related drugs [25]. Several case reports have demonstrated that some osimertinib-resistant *EGFR* mutations are responsive to the first and second-generation EGFR-TKIs [26–28] or combination therapies with osimertinib and gefitinib [29, 30]. To achieve extended overall life, the optimum regimen for overcoming TKI resistance must be developed.

In this work, we examined the transforming activity and TKI sensitivity of EGFR variants including very rare ones with unclear clinical relevance. We created heterogeneous tumors comprised of multiple EGFR variants, and treated them with EGFR-TKI combinations to assess the effectiveness of individual regimens with the mixed-all-nominated-in-one (MANO) method developed in our laboratory [31].

## Materials and methods

### Cell lines

Human embryonic kidney (HEK) 293 T cells and mouse 3T3 fibroblasts were obtained from the American Type Culture Collection (Manassas, VA, USA) and cultured in Dulbecco’s Modified Eagle’s medium-F12 (DMEM-F12) supplemented with fetal bovine serum (FBS), 2 mmol/L glutamine (Thermo Fisher Scientific, Waltham, MA, USA) and 1% penicillin/streptomycin (P/S). The Ba/F3 cells were cultured in the Roswell Park Memorial Institute (RPMI) 1640 medium (Thermo Fisher Scientific) supplemented with 10% FBS, 2 mmol/L glutamine, 1% P/S, and mouse IL-3 (20 U/mL; Sigma-Aldrich, St. Louis, MO, USA). Mouse 3T3 fibroblasts and Ba/F3 cells were used for the assessment of oncogenicity as 3T3 focus formation assay (FFA) is a conventional assay to evaluate the transforming potential of oncogenes [32], whereas IL-3-dependent Ba/F3 cell line is another popular system to screen for oncogenic variants [33]. The Ba/F3 system can also be used to investigate the susceptibility of driver variants to therapeutics [31, 34].

### Establishment of a retroviral vector with random barcodes

The pCX6 vector was established by inserting random 10-base pair (bp) DNA barcode sequences upstream of the start codon of the genes of interest into the pCX4 vector [35] (Supplementary Table 1). Next, the full-length wild-type cDNAs of human EGFR and control genes were inserted into the pCX6 vector. A total of 357 EGFR variants with at least two cases reported in the COSMIC v81, a database for somatic mutations in cancer, were selected in this study. All plasmids encoding EGFR variants were constructed by the GeneArt Gene Synthesis system (Thermo Fisher Scientific) and sequenced by next-generation sequencing to confirm. Three clones (some being one or two) with individual barcodes were constructed for each variant to obtain triplicate data for each assay.

### Creation of retrovirus and infection into cell lines

The recombinant plasmids were transduced with the packaging plasmids (Takara Bio, Shiga, Japan) into HEK293T cells in order to produce recombinant retroviral particles. The 3T3 cells were infected with ecotropic recombinant retroviruses in 96-well plates by using 4 μg⁄mL Polybrene (Sigma-Aldrich) for 24 h. The Ba/F3 cells were seeded on the RetroNectin-coated (Takara Bio) 96-well plates and infected with retroviruses in the RPMI 1640 medium supplemented with 20 U/mL of IL-3.

### FFA and scoring transforming activity

To evaluate the anchorage-independent growth, the 3T3 cells expressing EGFR variants were cultured in DMEM-F12 supplemented with 5% bovine calf serum for 2 weeks, and the cells were stained with the Giemsa solution. The transforming activity of each variant was measured by the FFA score. This score classified the focus-forming potential into the following four score groups: score 1 (negative), with no focus formation; score 2 (mild), partial focus formations of transformed cells; score 3 (moderate), diffusely transformed cells were piled up in bundles; score 4 (severe), round-shaped and anchorage-independent focuses were diffusely observed.

### The MANO method

A schema of the MANO method is depicted in Supplementary Fig. 1. In this method, a retroviral vector can stably integrate individual genes into the genome of assay cells along with 10-bp barcode sequences. Individually transduced assay cells were collected and cultured competitively to assess their transforming potential or drug sensitivity. Genomic DNA was extracted from the cell lysates by using the QIAamp DNA Mini Kit (Qiagen, Hilden, Germany), followed by amplification by polymerase chain reaction (PCR) with primers (Supplementary Table 2), including indices and adaptor sequences of Illumina. The sequencing libraries were prepared using the NEBNext Q5 Hot Start HiFi PCR Master Mix (New England Biolabs, Ipswich, MA, USA) according to the manufacturer’s instructions, and the obtained products were purified using the AMPure Beads (Beckman Coulter, Brea, CA, USA). The quality of the libraries was assessed using the Qubit 2.0 Fluorometer (Thermo Fisher Scientific) and the Agilent 2200 TapeStation System (Agilent Technologies, Santa Clara, CA, USA). The libraries were sequenced on the Illumina MiSeq by using the Reagent Kit V2 (300 cycles), and 150-bp paired-end reads were created (Supplementary Table 3). The barcode sequence 5′-CTAGACTGCCXXXXXXXXXXGGATCACTCT-3′ (where X denotes any nucleotide) was included in the sequencing results, and the number of barcodes in each variant was then quantified.

### Cell growth competition assay

The Ba/F3 cells expressing EGFR variants were cultured in the RPMI 1640 medium without IL-3. The transformed Ba/F3 cells that showed IL-3-independency were mixed on day 0 and then passaged on days 2, 4, and 7. The relative cell proliferation of variants was evaluated by the MANO method.

### Drug sensitivity assay using the MANO method in vitro

The transformed Ba/F3 cells were mixed in equal amounts and then treated with the indicated concentrations of afatinib (500 pM–50 nM), osimerinitib (100 pM–10 µM), and 10% dimethyl sulfoxide (DMSO, as the vehicle control) for 4 days. EGFR-TKIs used in the assays were purchased from LC Laboratories, Woburn, MA, USA. The experiment was conducted in both biological and technical triplicate. The number of each barcode was calculated by using the MANO method. Cells expressing the variant of KRAS G12V were used as the reference control for scaling the barcode count of each variant. The relative growth inhibition of each variant was then calculated as the ratio of the average read number across triplicates to that of the control. The R package SynergyFinder and the Bliss synergy model were used to calculate the drug synergy score [36]. The score at each concentration was calculated and the average of the scores for all concentrations was calculated.

### Tumor xenograft assay

In this study, animal assays were conducted according to the protocols approved by the Animal Ethics Committee of the National Cancer Research Center (Tokyo, Japan). In the first pilot study, the 3T3 cells expressing 22 EGFR variants (1.0 × 10^5^ for each) were mixed and injected into 6-week-old female nude mice. Gefitinib, afatinib, and/or osimertinib were dissolved in 10% DMSO, 10% 2-hydroxypropyl-beta-cyclodextrin, and sterile ultrapure water. Each group of 21 mice was treated with EGFR-TKI or the vehicle for 25 days (the drugs were switched on day 15) and the tumor volume was calculated using the following formula: *π*/6 × (large diameter) × (small diameter)^2^. EGFR-TKI were treated with oral administration of gefitinib (10 mg/kg body weight), afatinib (10 mg/kg body weight), or osimertinib (20 mg/kg body weight). Three mice were killed on days 5, 9, 13, 15, 17, 21, and 25. In the second experiment, the 3T3 cells with 31 EGFR variants (1.0 × 10^5^ for each) were mixed and injected into the mice, and the mice were then treated with gefinitib, afatinib, and/or osimertinib. The drugs were switched on day 16. Each group of 21 mice was treated for 30 days after cell injection and the tumor volumes were calculated every day. The mice were killed on days 7, 10, 13, 16, 19, 24, and 30. In the third experiment, where 357 variants of *EGFR* were used, a total of 1.25 × 10^6^ of the 3T3 cells were mixed, and injected into an individual mouse. Each group of 10 mice was treated with afatinib and/or osimertinib, and the tumor volumes were calculated on days 10, 13, 15, 18, and 20. The mice were killed on days 15 and 21. In these experiments, the resected tumors were manually subdivided into 2–4-mm squares by a scalpel, and tissue dispersal was performed by using Tumor Dissociation Kit, mouse, and gentleMACS Octo Dissociator with Heaters (Miltenyi Biotec, Bergisch Gladbach, Germany). DNA of the tumor was extracted using the QIAamp DNA Mini Kit (Qiagen), and the relative abundance of each clone compared with the green fluorescent protein cell clone was determined by using the MANO method. Significant differences were assessed using paired *t* test.

### PrestoBlue cell viability assay

The transformed Ba/F3 cells expressing each EGFR variant were cultured into 96-well plates (with 100 µL of culture medium per well) in the RPMI 1640 medium without IL-3, whereas afatinib (50 pM–10 nM) and/or osimerinitib (100 pM–1 µM) was added at different concentrations and treated for 4 days. Then, 10 μL of PrestoBlue (Thermo Fisher Scientific) was added to the plates, and the fluorescence was measured (at excitation 530 nm, emission 590 nm) after 3 h of incubation at 0.1 s.

## Results

### Transforming ability of EGFR variants in vitro

**Fig. 1:**
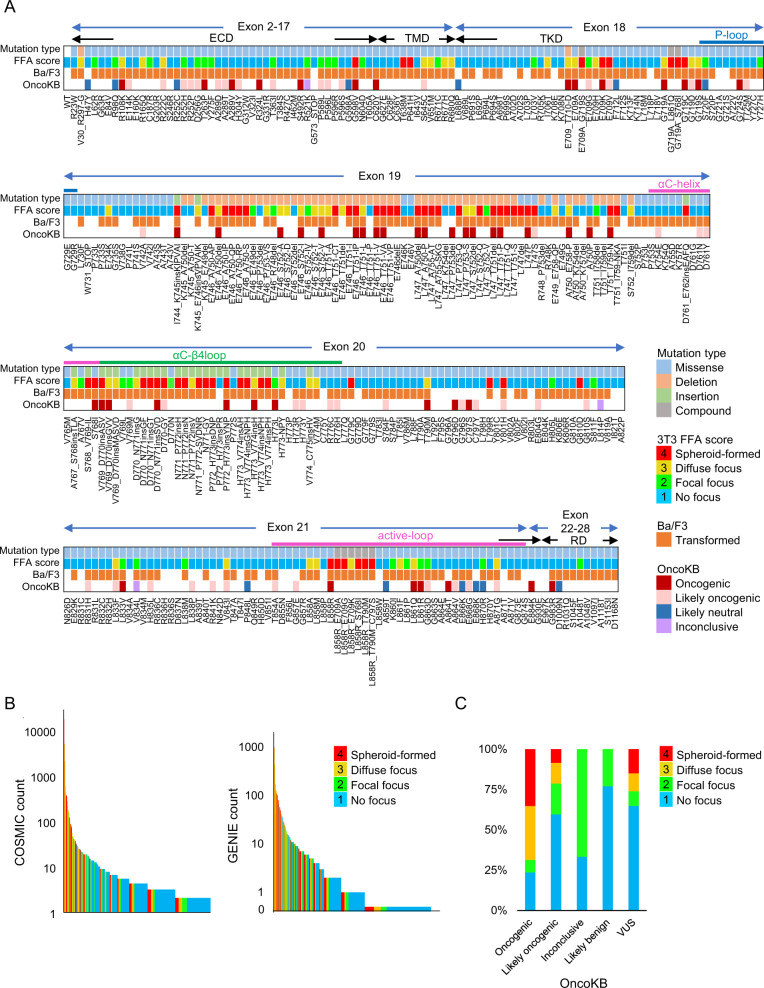
Summary of the transforming potential of 357 EGFR variants by the focus formation assay. **A** Information of each variant. Mutation type, FFA scores, transformation in the Ba/F3 cells, and annotation of OncoKB are indicated. The domain names are shown in the top panel. ECD, extracellular domain; TMD, transmembrane domain; TKD, tyrosine kinase domain; RD, regulatory domain. **B** The relationships between the FFA score and the variant count number of the COSMIC database or the GENIE cohort. Variants are arranged in the descending order of the count number. **C** Comparison of the FFA scores with OncoKB annotation. The ratio of FFA score in each group as defined by the annotation of OncoKB is shown. A variant of unknown significance indicates that the variants are not listed on OncoKB.

The 3T3 FFA was used to assess the transforming potential of 357 EGFR variants. As shown in Fig. 1A and Supplementary Fig. 2, 29 variants (8.1%) were assigned FFA score 2 (mild transformation), 50 (14.0%) were assigned FFA score 3 (moderate transformation), 65 (18.2%) were assigned FFA score 4 (severe transformation), and the remaining 213 variants (59.7%) were assigned FFA score 1 (no transformation). About half of the variants in exon 19 or 20 had a score of 2 or higher, while the majority of variants within exon 22–28 had a score of 1.

The transforming activity of EGFR variants was also examined in an IL-3–dependent pro-B cell line, Ba/F3, and 282 variants (79.0%) could confer IL-3-independent growth to Ba/F3. The growth competition assay of Ba/F3 cells revealed that exon 20 insertion variants stimulated cell proliferation more strongly than variants of the other exons (Fig. 1A and Supplementary Fig. 3).

The transforming assay results are summarized in Fig. 1A and Supplementary Table 4 with variant information such as variant type, exon number, domain category, and OncoKB annotation. Variants with a high COSMIC and GENIE count are more likely to have a high transforming potential (Fig. 1B). In this study, 26 variants with FFA scores of 3 or 4 were identified as oncogenic, which were not previously reported in OncoKB (Fig. 1C). The majority of them were in exon 19 or 20.

### In vitro drug sensitivity of EGFR variants to EGFR-TKIs

**Fig. 2:**
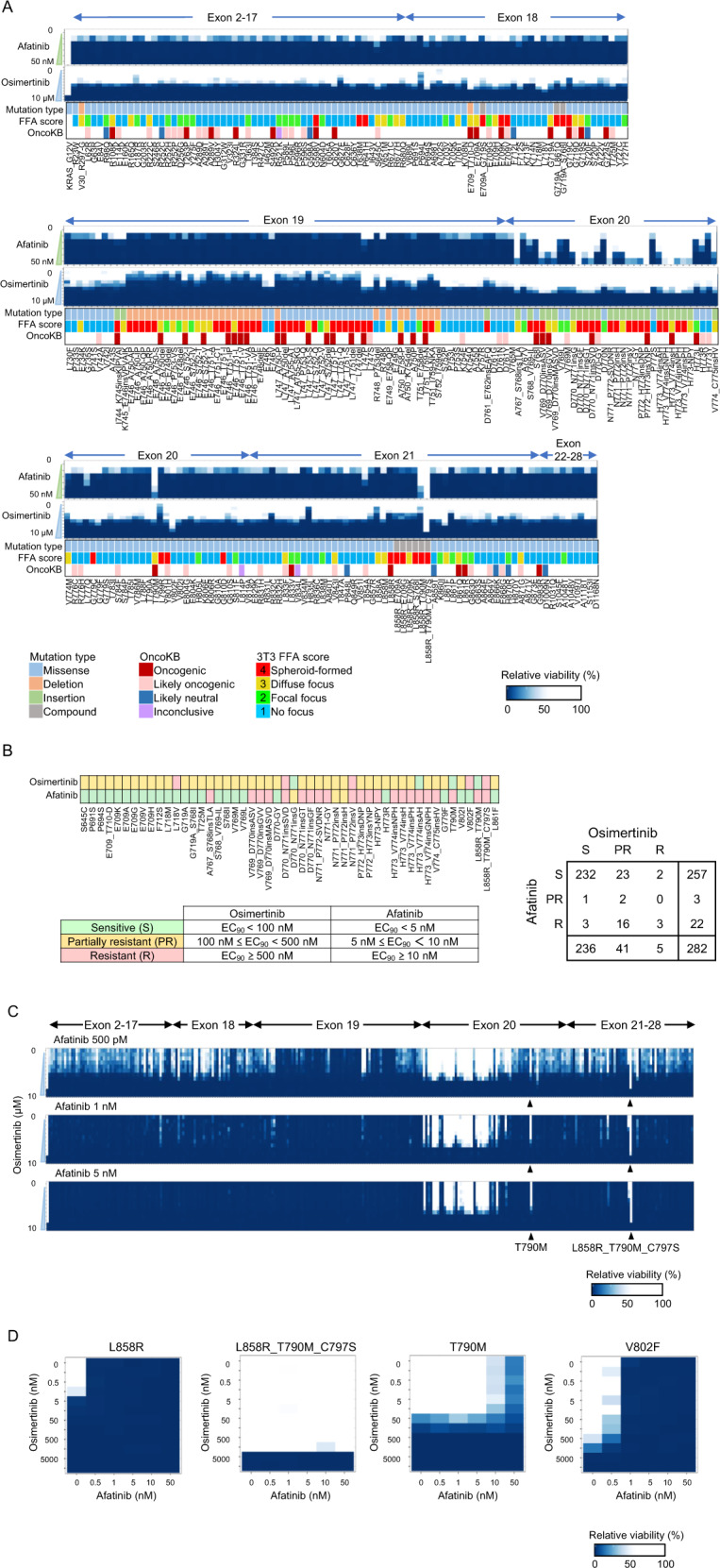
Drug sensitivity of 282 EGFR variants by the MANO method in vitro. An overview of drug sensitivity of the EGFR variants. The Ba/F3 cells expressing EGFR variants were treated with afatinib and osimertinib at the indicated concentrations. **A** Cells were treated with afatinib or osimertinib. KRAS_G12V is shown in the far left panel as the control. Lower bands indicate information about the mutation type, FFA score, and OncoKB annotation. **B** Variants that were resistant or partially resistant to afatinib and/or osimertinib are shown. **C** Drug sensitivity of the combination treatment. Details of the drug sensitivity data of each variant are provided in Supplementary Table 5. The enlarged figure of the results of variants of exon 20 with the combination therapy is shown in Supplementary Fig. 4A. **D** Drug sensitivity of each variant. The concentrations of afatinib and osimertinib are indicated on the *X* and *Y* axis, respectively. The figures of the representative variants exhibiting four different drug sensitivities are presented. L858R showed sensitivity to both the drugs, L858R_T790M_C797S showed resistance to both the drugs, L858R_T790M showed resistance to afatinib only, and V802F showed resistance to osimertinib only.

To assess the drug sensitivity of EGFR variants, transformed Ba/F3 cells expressing 282 EGFR variants were treated with afatinib or osimertinib using the MANO method (Fig. 2A and Supplementary Table 5). TKI-sensitizing mutations such as exon 19 deletions and L858R were found to be sensitive to afatinib and osimertinib, whereas TKI-resistant mutations including exon 20 insertions and L858R_T790M_C979S were found to be resistant to both TKIs, indicating the feasibility of the MANO method in drug sensitivity assays with this scale.

Out of the 282 variants, 50 (17.7%) were found to be resistant or partly resistant to osimertinib or afatinib (Fig. 2B). Three D770_N771insSVD, N771_P772insV, and L858R_T790M_C797S variants (1.1%) were found to be resistant to both TKIs, whereas 21 variants (7.4%) were found to be nonsensitive to both TKIs. Importantly, among 25 variants that were refractory to afatinib, four (16%) were sensitive to osimertinib, whereas 25 of 46 osimertinib-resistant variants (54.3%) were sensitive to afatinib.

The MANO method was then used to assess the drug combination sensitivity of 282 EGFR variants to afatinib and osimertinib in transformed Ba/F3 cells expressing EGFR variants (Fig. 2C, Supplementary Fig. 4 and Supplementary Table 5).

In a two-dimensional heat map, the sensitivity of each variant to the combination therapies was precisely depicted (Fig. 2D). The variants were classified into four groups based on the drug sensitivity to afatinib and osimertinib: variants sensitive to both drugs such as L858R, variants resistant to both drugs such as L858R_T790M_C797S and V769_D770insASV (exon 20 insertions), variants sensitive to osimertinib but resistant to afatinib such as T790M, and variants sensitive to afatinib but resistant to osimertinib such as V802F and L718V. Supplementary Fig. 5A–C shows heat maps of additional variants that are resistant or partly sensitive in vitro. Afatinib with osimertinib had no significant synergistic impact (Supplementary Fig. 6).

### Evaluation of EGFR-TKI treatments in vivo

The 3T3 cells expressing 22 EGFR variants were implanted into mice to study the therapeutic impact of TKIs against heterogeneous tumors consisting of numerous *EGFR* mutations. Classical type EGFR variants (such as L858R and exon 19 deletion), uncommon variants (such as G719X, E709X, S768I, L861Q, and exon 20 insertion), and a resistant variant to first- and second-generation EGFR-TKIs (T790M) were selected. Mice with the inoculated tumors were given afatinib for 10 days before switching to osimertinib (regimen AO) or given gefitinib for 10 days before switching to osimertinib (regimen GO) (Supplementary Fig. 7A). TKI therapies inhibited tumor development as compared with vehicle; however, there was no significant difference in the tumor size between AO and GO (Supplementary Fig. 7B).

The tumors were excised every 2–4 days to track intratumoral variant composition using the MANO method. After 20 days, cells with D770_N771insSVD or D770_N771insG accounted for more than 80% of the total number (Supplementary Fig. 7C), far exceeding the other variants. This finding revealed that tumors with exon 20 insertions were particularly resistant to EGFR-TKIs, and developed quickly in comparison to the other variants.

### Differences in the efficacy between each combination treatment

**Fig. 3:**
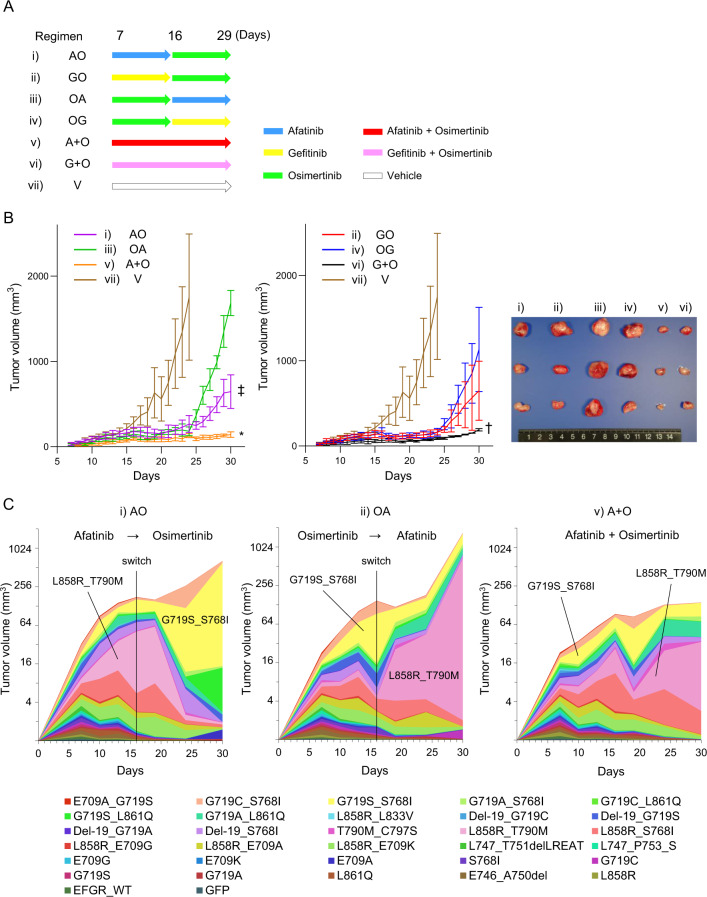
Comparative experiments on the drug administration methods. **A** A schema of the treatment regimens employed in the study. **B** The tumor growth of each group is shown in the left panel. The images of tumors resected on day 30 from the indicated groups are shown in the right panel. The tumor size of the A + O group was significantly smaller than that of the AO, OA, and OG groups at day 30 (*p = 0.023 vs AO, *p* < 0.01 vs OA, *p* = 0.047 vs OG). The tumor size of the G + O group was significantly smaller than that of the AO and OA groups (^†^*p* = 0.031 vs AO, *p* < 0.01 vs OA). The tumor size of the OA group was significantly larger than that of the AO and GO groups (^‡^*p* = 0.004 vs AO, *p* = 0.030 vs GO). The tumor volume is shown as a mean with SD (error bars). **C** Changes in the proportion of variants were evaluated by the MANO method.

We then compared the in vivo efficacy of several treatment regimens for EGFR-TKIs. For this experiment, we removed the exon 20 insertion mutants to keep a heterogeneous tumor model comprised primarily of TKI-sensitive EGFR variants (Fig. 3A). The six regimens were as follows: AO (starting with afatinib followed by osimertinib), GO (starting with gefitinib followed by osimertinib), OA (starting with osimertinib followed by afatinib), OG (starting with osimertinib followed by gefitinib), A+O (a combinational therapy of afatinib and osimertinib) and G+O (a combinational therapy of gefitinib and osimertinib).

Strong tumor growth suppression was observed in the A+O and G+O groups. The A+O group had considerably smaller tumors than the AO, OA, and OG groups at day 30 (*p = 0.023 vs AO, *p* < 0.01 vs OA, and *p* = 0.047 vs OG). The tumor size in the G+O group was substantially smaller than in the AO and OA groups (^†^*p* = 0.031 vs AO, *p* < 0.01 vs OA).

Regimens that began with the first- or second-generation TKIs, such as AO or GO, tended to inhibit tumor development more successfully than those that began with the third-generation TKIs, such as OA or OG. The tumor size of the AO group was significantly smaller than that of the OA group (^‡^*p* = 0.004), whereas the tumor size of the GO group did not differ significantly from that of the OG group (*p* = 0.321) (Fig. 3B).

The MANO method was used to analyze the temporal changes of each variant fraction (Fig. 3C and Supplementary Fig. 8). During the AO regimen, the L858R_T790M variant grew to occupy 50% of the tumor during the afatinib therapy and then diminished following the commencement of osimertinib. G719S_S768I and G719S_L861Q, on the other hand, rose once the therapy was changed to osmiertinib. In the OA regimen, the reverse pattern was seen, with L858R_T790M increasing to almost 80% when the therapy was switched to afatinib. The ultimate tumor size of the AO regimen was considerably less than that of the OA regimen.

Certain variants, notably L858R_T790M, grew slowly but steadily under the A+O regimen. Changes in the cell fraction with each variant in the gefitinib regimen were nearly identical to those in the afatinib regimen (Supplementary Fig. 8). This is most likely due to the similarities in the targetable variants of first- and second-generation EGFR-TKI.

### Assessment of drug sensitivity of the 336 EGFR variants in vivo

**Fig. 4:**
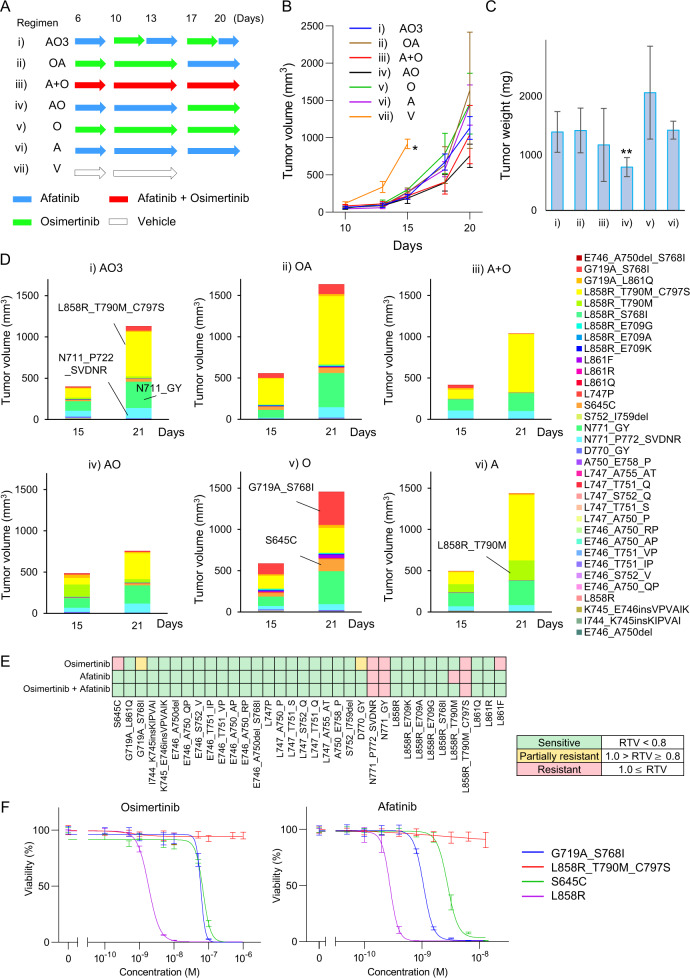
Drug sensitivity experiments using 6 drug administration types and 336 variants of EGFR. **A** A schema of drug administration schedule. Half of the mice were killed on day 15 and the other half on day 21. **B** Changes in the tumor volumes are estimated by measuring the tumor diameters. In the vehicle group, mice were killed as their tumor volume reached 100 mm^3^ on day 15. The tumor volume is shown as a mean with SD (error bars). **C** Tumor weight was measured on day 30. The tumor weight is shown as a mean with SD (error bars). **D** Graphs indicate the percentages of variants on days 15 and 21. Only the 34 variants with a sufficient number of barcode reads are shown. **E** In vivo, the drug sensitivity of 34 variants was assessed. The relative tumor volume (RTV) of each variant was compared with that of the vehicle group on day 15. **F** The results of cell viability assay using osimertinib and afatinib. The cell viability is shown as mean with SD (error bars).

To better understand the mechanism of drug resistance in heterogeneous tumors in vivo, we generated 3T3 cells each carrying 336 EGFR variants that were injected en bloc into mice. For this experiment, the major 21 variants of exon 20 insertion were excluded from the pool. A total of seven medication regimens were begun on those mice on day 6 following the injection, and tumor volume was assessed on days 10, 13, 15, 18, and 20. (Fig. 4A).

The tumor volume of the AO group was substantially lower than that of the AO3 (3-day alternating therapy with afatinib and osimertinib), O, and A groups (*p = 9.4 × 10^−3^ vs AO3, *p* < 0.012 vs O, *p* = 4.8 × 10^−3^ vs A) (Fig. 4B). The tumor weight of the AO group was substantially lower than that of the AO3, OA, O, and A groups (**p = 0.014 vs AO3, *p* < 0.017 vs OA, *p* = 0.014 vs O, *p* < 0.01 vs A) (Fig. 4C).

Tumors were resected on days 15 and 21 to assess the proportions of cells with EGFR variants using the MANO method (Fig. 4D and Supplementary Fig. 9). Regardless of medication regimens, variants of L858R_T790M_C797S, N771_GY, and N771_P772_SVDNR have shown a high cell proportion in all groups. On day 21, tumors treated with osimertinib were mostly consisted of seven variants (variant allele frequency >1.0%), whereas tumors treated with afatinib were primarily made of four variants, implying that tumors treated with osimertinib were heterogeneous, consisting of numerous variants. In this group, a significant increase of S645C and G719A_S768I was detected, indicating resistance to osimertinib.

Furthermore, on day 15, the relative tumor sizes of individual variants treated with different regimens were compared with those treated with a vehicle to determine the drug sensitivities of the cells expressing 34 variants. As shown in Fig. 4E, S645C, G719A_S768I, D770_GY, N771_P772_SVDNR, N771_GY, L858R_T790M_C797S, and L861F were resistant or partially resistant to osimertinib. In the in vivo assessment of 34 variants, 32 variants (94.1%) were consistent with the in vitro sensitivity experiment in Fig. 2B.

An in vitro cell viability experiment was conducted to validate the resistant profile of S645C and G719A S768I to osimertinib (Fig. 4F). The IC_50_ values for S645C and G719A_S768I to osimertinib were 69 nM and 59 nM, respectively, whereas those for afatinib were 2.7 nM and 1.1 nM.

## Discussion

We were able to explore the in vitro drug sensitivity of 282 EGFR variants to afatinib or osmiertinib as a monotherapy as well as the combination treatment of afatinib and osimertinib. The combinational therapy was carried out at 72 distinct concentrations for each compound, producing almost 20,000 drug treatment data. This is the first study to look at a combination medication therapy for this many *EGFR* mutations.

The MANO method’s drug sensitivity study was consistent with prior clinical and pre-clinical findings on well-known variants such as L858R, exon 19 deletions, L858R_T790M, and L858R_T790M_C797S. The drug spectrums of afatinib and osimertinib were shown to be distinct. Although there is no substantial synergistic impact of afatinib and osimertinib, most EGFR variants, except for exon 20 insertions and L858R_T790M_C797S variants, could be treated with at least one of the medicines, indicating the viability of combination therapy.

The MANO approach revealed that exon 20 insertions are less susceptible to afatinib and L718V, L802F variants less sensitive to osimertinib, which was consistent with prior studies [37–39]. Furthermore, S645C and G719A/S_S768I were shown to be osimertinib-resistant in vivo. S645C has been linked to tumor formation [40], whereas G719A_S768I is particularly sensitive to afatinib [41–43]. Different sensitivities of the same exon variants are probably due to changes in the orientation of the molecular structure [44]. More research is needed to assess the sensitivities of osimertinib-resistant mutations to other third-generation EGFR-TKIs presently under development.

The heterogeneous tumor model provided some crucial findings. First, as shown in Fig. 3, successful combination therapy was observed if the tumor had only a few clones that were resistant to both medicines. In contrast, combined therapies were ineffective if a tumor included a significant number of resistant clones to both medicines, as seen in Fig. 4 and Supplementary Fig. 7. Given that primary NSCLC with a TKI-sensitizing EGFR-mutant may not harbor resistant clones at the start of TKI treatment, we anticipate that combination therapy with afatinib and osimertinib may improve patient prognosis by reducing the likelihood of the resistant clone appearing, which is supported by previous research [45, 46].

Second, inhibiting the tumor’s main clone is critical for tumor progression management. When treatments were shifted from afatinib to osimertinib, cell growth was markedly repressed; most likely because the dominant clone of L858R_T790M was depleted, resulting in tumor growth reduction as a whole. This might explain why regimens beginning with afatinib and ending with osimertinib suppressed tumor development more effective than the opposite combination.

Third, even with a treatment-sensitive mutation, it may be difficult to eliminate tumor cells completely. Surprisingly, the L858R_T790M mutation tolerated osimertinib therapy and grew after switching to afatinib. Therefore, in clinical practice, it may be beneficial to monitor mutations using liquid biopsy regularly and switch to the best medication based on the remaining variants discovered.

Fourth, the model used in this study might be applied to clinical combinational therapy regimens. The following clinical trials are, for instance, currently underway; FLAURA2 (NCT04035486), a phase III study of osimertinib with or without platinum plus pemetrexed chemotherapy; MARIPOSA (NCT04487080), a phase III trial study of amivantamab and lazertinib combination therapy versus osimertinib; ACROSS1 (NCT04500704) and ACROSS2 (NCT04500717), phase III trial studies of almonertinib alone versus almonertinib plus chemotherapy.

Given that exon 20 insertions and L858R_T790M_C797S are resistant to both TKIs, new drug development is eagerly anticipated. Regardless of the underlying mutations, amivantamab has proven efficacy in EGFR mutation-positive NSCLC (deletions within exon 19, L858R, L858R_T790M, L858R_T790M_C797S, exon 20 insertions). This new EGFR/MET bispecific monoclonal antibody has the potential to provide a therapeutic efficacy by inhibiting the rise of resistant mutants to EGFR-TKIs [47].

With regard to the concordance between in vitro and in vivo assays, variants grew strongly in the in vivo tumor model, such as L858R_T790M, G719S_S768I, G719C_L861Q, L858R_S768I in Fig. 3C, and L858R_T790M_C797S, N711_GY, N711_P722_SVDNR, S645C, G719A_S768I in Fig. 4D were less sensitive to the respective drugs (Fig. 2) and tended to higher transforming ability in vitro (Fig. 1).

There are following limitations to this study. First of all, throughout the initial in vivo evaluation, several variants were drained from the pool by competitive outgrowth of other variants. To maximize the number of variants for evaluation, EGFR variants should be categorized into three to four groups based on their transforming activity. In previous work, for example, a relatively small-scale in vivo MANO method was effectively used to analyze exon 20 insertions with high growth potential [34]. Second, this study uses mouse cells to investigate the human gene variants. Therefore, the results may not be the same in the human cells. Especially, the tumor growth with the 3T3 cell model is very aggressive and too rapid to accurately assess their responses to the various regimens in vivo. In the experiment on Fig. 4, the tumor growth was so fast that the mice had to be killed on day 21, which was earlier than we originally planned (day 30). Third, our analysis did not account for additional genetic changes such as *PIK3CA* mutations or *MET* amplification which can co-occur in tumors with *EGFR* mutations and may impact treatment sensitivity. Fourth, while this pre-clinical investigation suggested that utilizing a combination of TKIs might be beneficial, the adverse effects of taking the two medicines were not explored. More experiments and clinical trials must be used to address these constraints.

In conclusion, the transforming capacity and drug sensitivity of EGFR variants to EGFR-TKIs were thoroughly evaluated. It has been proposed that EGFR-TKI combination therapy may limit tumor development and regulate the acquisition of resistant variants. In clinical practice, it is critical to choose medicines that target given variants in patients. The MANO method used in this study can be further extended to various types of combination medicines. This study’s findings might serve as a foundation for optimizing combinational treatments and promoting the creation of ideal regimens to enhance patient prognosis.

## Supplementary information

Supplementary figures 1–9

Supplementary Table 1

Supplementary Table 2

Supplementary Table 3

Supplementary Table 4

Supplementary Table 5
