# Supplementary material for: Preclinical assessment of combination therapy of EGFR tyrosine kinase inhibitors in a highly heterogeneous tumor model
Source: Oncogene. 2022 Mar 19;41(17):2470–9. doi: 10.1038/s41388-022-02263-4 (PMC9033582; doi:10.1038/s41388-022-02263-4)
Supplement: Supplementary file 1 — Supplementary figures 1–9 [file 41388_2022_2263_MOESM1_ESM.docx]

**Supplementary Figures**

**Preclinical assessment of combination therapy of EGFR tyrosine kinase inhibitors in a highly heterogenous tumor model**

**Authors:** Hiroshi Ikeuchi, Takeshi Hirose, Masachika Ikegami, Kazuya Takamochi, Kenji Suzuki, Hiroyuki Mano, and Shinji Kohsaka

**Contents:**

**Supplementary Figure 1–S9**

**Supplementary Figure 1. A schema of the MANO method.**

Retroviral vectors could stably integrate individual genes into the genome of 3T3 or Ba/F3 cells with barcode sequences. The cells were collected and cultured competitively to assess their transforming potential or drug sensitivity. Genomic DNA was extracted from cells treated with drugs and sequences. The number of barcodes in each variant was quantified. The details of the focus formation assay and the assessment of the FFA score are shown in the Materials and Methods.


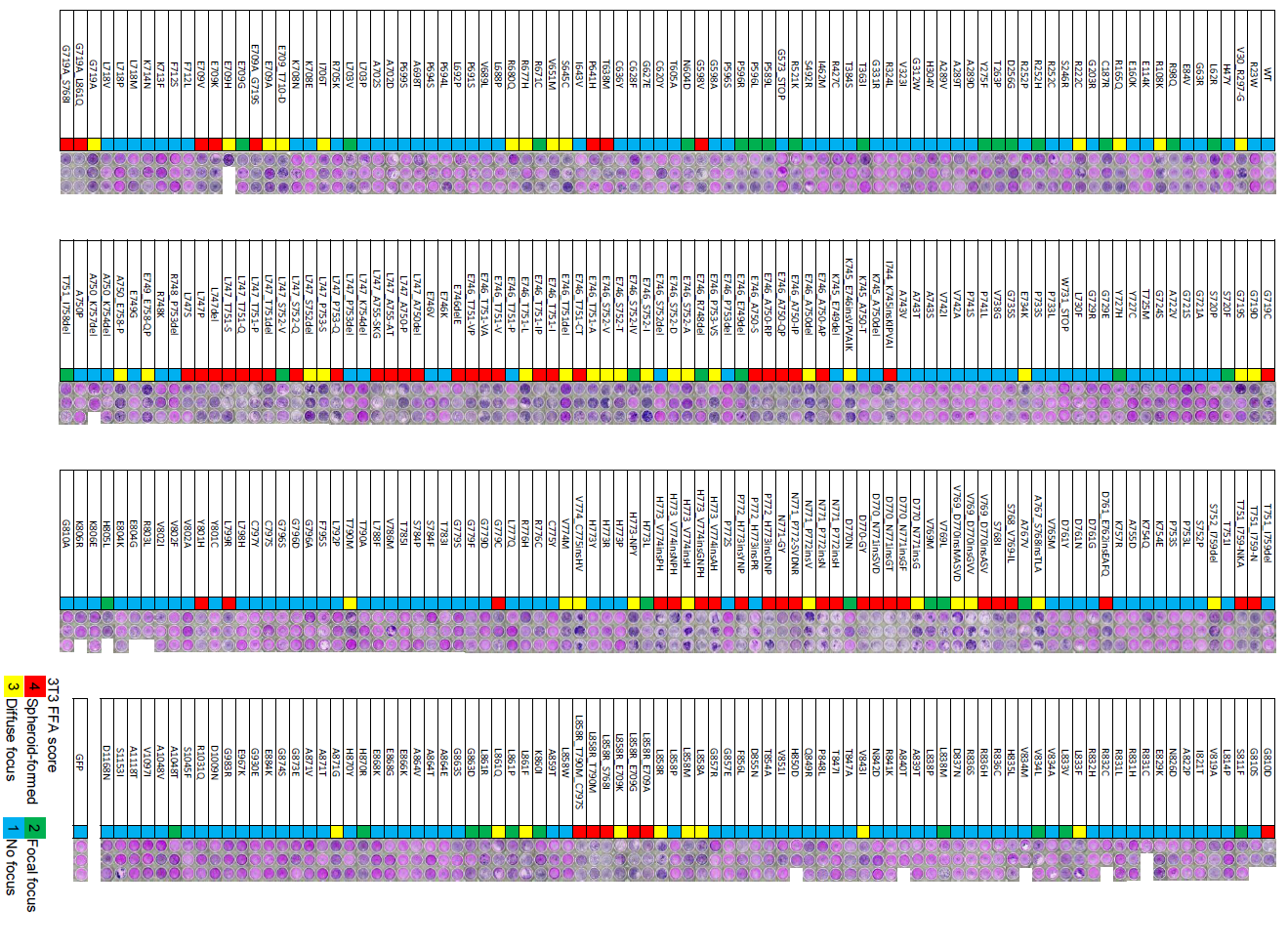


**Supplementary Figure 2. Image of the focus formation assay**

Images of the 3T3 cells expressing EGFR variants stained with Giemsa solution after the focus formation assay. The colors in the middle column indicate the FFA score for each variant. Each variant with three different barcodes was prepared for triplicate assays.


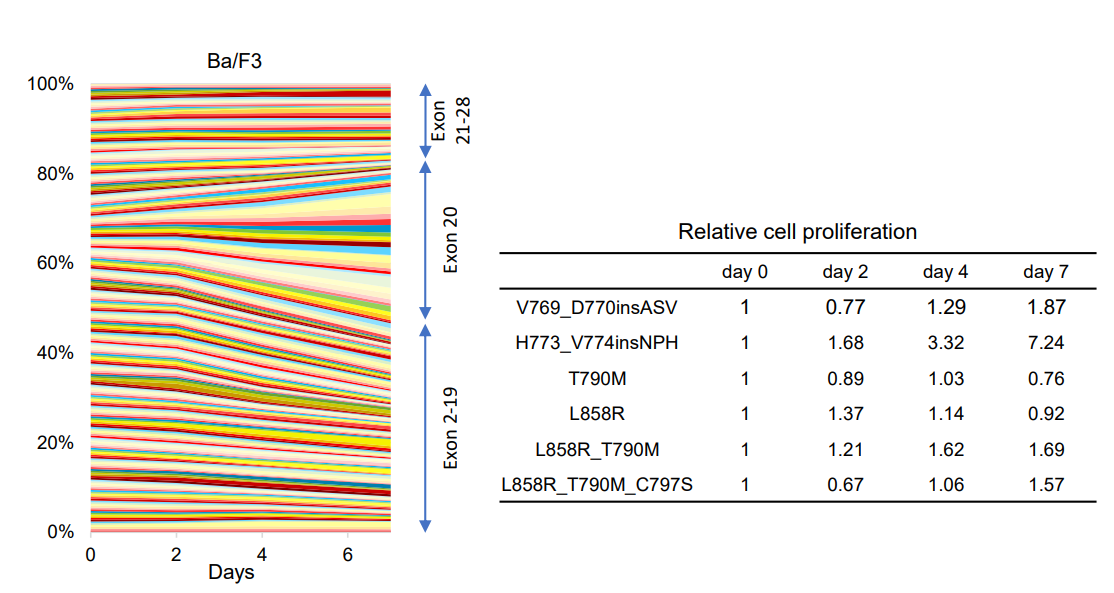


**Supplementary Figure 3. Cell growth competition assay using Ba/F3 cells**

A total of 255 EGFR variants with sufficient sequence reads were evaluated. Variants were arranged according to their amino acid position. Variants of exon 20 insertion showed strong growth activity. The table shows the relative cell proliferation of the common variants.


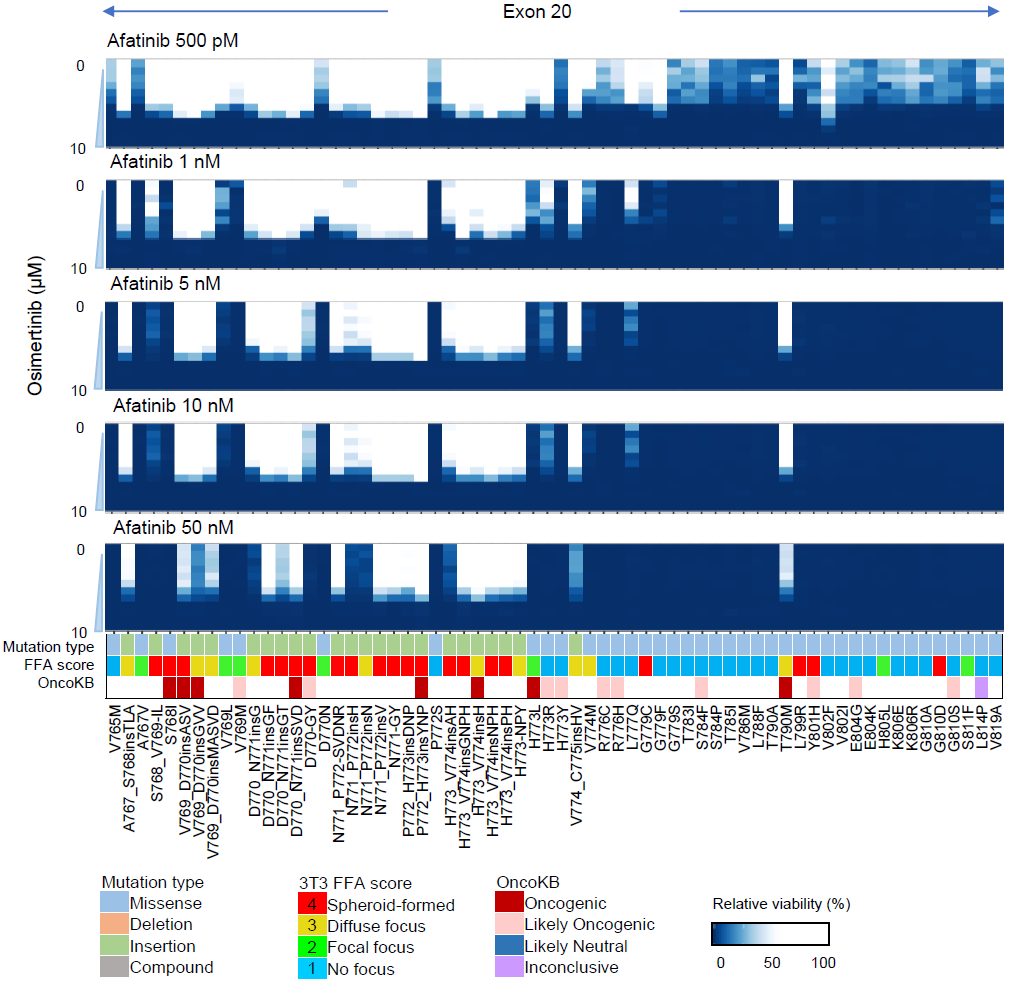


**Supplementary Figure 4. Combination drug sensitivity for each EGFR variant using Ba/F3 *in vitro*.**

Enlarged image of **Fig. 2C** showing the detailed combination drug sensitivities of exon 20 insertions.

**Supplementary Figure 5. The heat map patterns of combination drug sensitivity of variants resistant or partially resistant *in vitro.***

The variants were classified into four patterns based on drug sensitivity to afatinib and osimertinib: variants sensitive to both the drugs such as L858R shown in **Fig. 2D**, **(A)** variants resistant to both the drugs such as L858R_T790M_C797S and V769_D770insASV (exon 20 insertions), **(B)** variants sensitive to Osimertinib, but resistant to afatinib such as T790M and L858R_T790M, and **(C)** variants sensitive to afatinib, but resistant to osimertinib such as V802F and L718V.


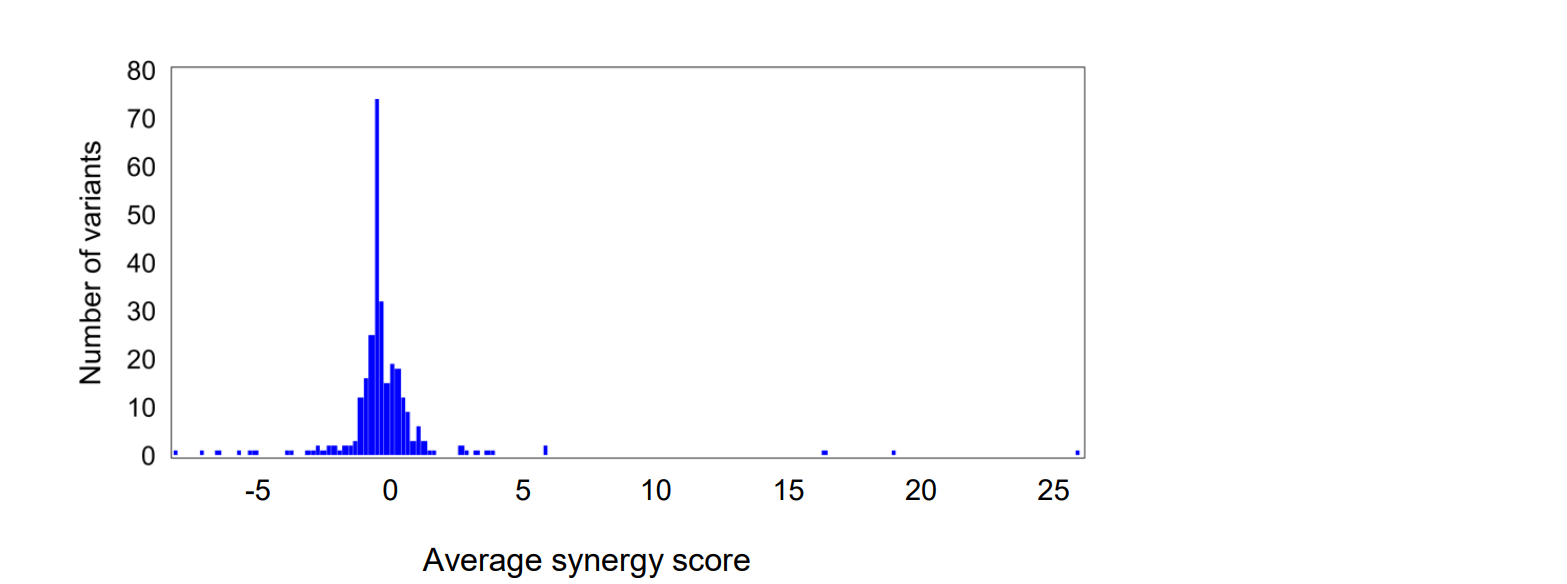


**Supplementary Figure 6. Average of synergy score for each variant**

The average synergy scores were calculated as indicated in the methodology section. The synergy score reveals the magnitude of the synergistic effect. 277 variants (98.2%) scored less than 5, which suggests that there is no synergistic effect between the two EGFR-TKIs.

**Supplementary Figure 7. Evaluation of the EGFR-TKI treatment *in vivo***

**(A)** Schema of the drug treatment schedule. **(B)** The tumor volume of each group is shown in the left panel. The images of tumors resected on day 25 from a group of AO (i) or GO (ii) are shown in the right picture. The tumor volume of the vehicle-treated group was significantly larger than those obtained in the AO and GO groups on day 17 (*, *p* < 0.01 vs AO and GO). The tumor volume is shown as mean with SD (error bars). **(C)** Changes in the proportion of variants were evaluated by the MANO method.

**Supplementary Figure 8. Changes in the proportion of EGFR variants treated with gefitinib and osimertinib**

Changes in the proportion of variants evaluated by the MANO method.

**Supplementary Figure 9. The evaluation of drug sensitivity of EGFR variants against six drug regimens.**

The relative viability of EGFR variants is indicated for six drug regimens. The relative cell viability of the individual variant was calculated by comparing with the bar code numbers of the variant in the vehicle-treated group and compensated with the value of L858R_T790M_C797S, a well-known drug-resistant variant. The 34 variants whose bar codes were > 300 in the vehicle group were analyzed. The relative cell viability is shown as mean with SD (error bars).
